# Rate and success of study replication in ecology and evolution

**DOI:** 10.7717/peerj.7654

**Published:** 2019-09-10

**Authors:** Clint D. Kelly

**Affiliations:** Département des Sciences biologiques, Université du Québec à Montréal, Montréal, Quebec, Canada

**Keywords:** Study replication, Effect size

## Abstract

The recent replication crisis has caused several scientific disciplines to self-reflect on the frequency with which they replicate previously published studies and to assess their success in such endeavours. The rate of replication, however, has yet to be assessed for ecology and evolution. Here, I survey the open-access ecology and evolution literature to determine how often ecologists and evolutionary biologists replicate, or at least claim to replicate, previously published studies. I found that approximately 0.023% of ecology and evolution studies are described by their authors as replications. Two of the 11 original-replication study pairs provided sufficient statistical detail for three effects so as to permit a formal analysis of replication success. Replicating authors correctly concluded that they replicated an original effect in two cases; in the third case, my analysis suggests that the finding by the replicating authors was consistent with the original finding, contrary the conclusion of “replication failure” by the authors.

## Introduction

Replicability is the cornerstone of science yet its importance has gained widespread support only in recent years (Kelly, 2006; Nakagawa & Parker, 2015; Mueller-Langer et al., 2019; Open Science Collaboration, 2015; Makel, Plucker & Hegarty, 2012). The “replication crisis” of the past decade has not only stimulated considerable reflection and discussion within scientific disciplines (Parker et al., 2016; Brandt et al., 2014; Kelly, 2006; Schmidt, 2009; Zwaan et al., 2017) but it has also produced large-scale, systematic efforts to replicate foundational studies in psychology and biomedicine (Iorns, 2013; Open Science Collaboration, 2015). Although behavioural ecologists have a poor track record of exactly replicating studies (Kelly, 2006), little is known about the extent to which the replication crisis plagues the broader community of studies in ecology and evolution. Indeed, evidence suggests that the issues causing low rates of replication in other scientific disciplines are also present in ecology and evolution (Fidler et al., 2017; Fraser et al., 2018).

There is much debate and confusion surrounding what constitutes a replication (Palmer, 2000; Kelly, 2006; Nakagawa & Parker, 2015; Brandt et al., 2014; Simons, 2014). Ecologists and evolutionary biologists frequently repeat studies using a different species or study system (Palmer, 2000). Palmer (2000) called this phenomenon “quasireplication”. Quasireplication differs from true replication in that the latter is, at its most basic level, performed using the same species to test the same hypothesis. There are three types of true replication (Lykken, 1968; Schmidt, 2009; Reid, Soley & Winner, 1981): exact, partial, or conceptual. Exact replications - also called direct, literal, operational, or constructive - generally entail some notion that the study is a duplication of another study (Schmidt, 2009; Simons, 2014). However, this is nearly impossible to achieve for obvious reasons (e.g., must use different pool of research subjects), and so most true replications are “close” or partial replications (Brandt et al., 2014). Partial replications involve some procedural modifications while conceptual replications (also called instrumental replication) test the same hypothesis (and predictions) using markedly different experimental approaches (Schmidt, 2009). It is useful to think of exact replications being at one end of the replication spectrum with quasi-replications at the other; partial and conceptual replications occupy the space between these extremes.

The replication of research studies (or at least the publication of replications) has been rather poor across disciplines in the social and natural sciences despite its need. For example, only 1% of papers published in the top 100 psychology journals were partial replications (Makel, Plucker & Hegarty, 2012) and estimates across other disciplines show equally low rates in economics (0.1%, Mueller-Langer et al., 2019), marketing (0%, Hubbard & Armstrong, 1994; 1.2%, Evanschitzky et al., 2007), advertising/marketing/communication (0.8%, Reid, Soley & Winner, 1981), education (0.13%, Makel & Plucker, 2014), forecasting (8.4%, Evanschitzky & Armstrong, 2010), and finance (0.1%, Hubbard & Vetter, 1991). Kelly (2006) found that 25–34% of the published papers in behavioural ecology’s top three journals (*Animal Behaviour*, *Behavioural Ecology and Sociobiology*, and *Behavioural Ecology*) were partial/conceptual replications whereas no exact replications were found.

Not only are scientists failing to conduct (or publish) replications, but more worryingly, we are failing to replicate original research findings when studies are repeated. Two separate studies conducted by The Many Labs project successfully replicated 77% (Klein et al., 2014) and 54% (Klein et al., 2018) of psychology studies. The Open Science Collaboration (2015) found that 36% of 100 studies in psychology successfully replicated. Other bioscience fields of research have shown equally poor replication success. For example, 11% of landmark preclinical cancer trials (Begley & Ellis, 2012) and 35% of pharmacology studies were found to replicate (Prinz, Schlange & Asadullah, 2011). Only 44% of the 49 most widely-cited clinical research studies replicated (Ioannidis, 2005). Similar rates are found in the social sciences as replications contradicted previously published findings in 60% of finance studies (Hubbard & Vetter, 1991), 40% in advertising, marketing and communication (Reid, Soley & Winner, 1981), and 54% in accounting, economics, finance, management, and marketing (Hubbard & Vetter, 1996). Camerer et al. (2016) found that 61% of laboratory studies in economics successfully replicated a previous finding.

Perhaps the apparent low success of replication studies stems from the manner in which success is judged. There is no single standard for evaluating replication success (Open Science Collaboration, 2015) but most replications are deemed successful if they find a result that is statistically significant in the same direction as the result from the original study (Simonsohn, 2015). This approach has several shortcomings (Cumming, 2008) not least of which is that our confidence in the original study is unnecessarily undermined when replications are underpowered (i.e., a low-powered, non-significant study calls the original finding into question) (Simonsohn, 2015). A second approach is to use meta-analytic techniques to assess whether the 95% confidence intervals of the original and replicate overlap. Rather than asking whether the replication differs from zero, this approach asks whether it differs from the original estimate. This method, however, is poor at detecting false-positives (Simonsohn, 2015). Simonsohn (2015) proposed an alternative approach based on the premise that if an original effect size was seen with a small sample size then it should also be seen with a larger sample size in a replicate study. If the effect size of the replicate study could not be detected with the sample size of the original study, then the effect is too small to have been reliably detected by the original experiment, and doubt is cast on the original observation.

We do not know if there is a ‘replication crisis’ in ecology and evolution. However, in order to make informed decisions on whether we need to change our views and behaviour toward replications, we need, as a first step, an empirical assessment of their frequency and success in the published literature. My aim in this paper is two-fold. First, I attempt to quantify the frequency of *Ecology, Evolution, Behavior, and Systematics* studies claiming to be true replications and then compare this rate with that of a general biology open access publication (*PeerJ*). Second, I calculate the success rate of replications found in these journals.

## Methods and Materials

On 4 June 2017, I downloaded as .xml files the 1,641,366 Open Access papers available in the PubMed database representing 7,439 journals. I then selected only those papers from journals categorized as *Ecology, Evolution, Behavior, and Systematics* within the “Agricultural and Biological Sciences” subject area of the SCImago Journal & Country Rank portal (https://www.scimagojr.com/). This resulted in a subset of 38,730 papers from 160 journals (see Supplemental Information for list of journals).

I used code written in the Python language (available at the Open Science Framework: DOI 10.17605/OSF.IO/WR286) to text-mine this subset of papers for any permutation of the word “replicate” (i.e., “replic*”) in the Introduction and Discussion (see also Head et al., 2015). For each instance of “replic*” I extracted the sentence as well as the paper’s meta-data (doi, ISSN, etc). Each of these instances was added as a row to a .csv file. I eliminated from this group papers published in *PLoS Computational Biology* because these studies did not empirically test ecological or evolutionary hypotheses with living systems. Text-mined papers were from non-open access journals (e.g., Animal Cognition) that provided an open access publishing option as well as open access journals (e.g., *Ecology & Evolution*). In order to compare rates of study replication in discipline-specific (i.e., *Ecology & Evolution*) open access (and hybrid) journals with a multidisciplinary open access journal I also text-mined 3,343 papers published in *PeerJ*.

I then included/excluded each paper based on whether the content of the extracted sentence dealt with a true replication (i.e., exact, partial or conceptual). I did not include quasireplications (Palmer, 2000) or studies that re-analyzed previously published data (e.g., Amos, 2009). Many articles that used the term “replic*” but were not true replications, instead using the term in the context of stating that the results needed to be replicated, explaining an experimental design (e.g., replicated treatments), or making reference to DNA studies (e.g., replicated sequences). If the article reported on a replicated study, I retrieved the original study and the replication from the literature. By reading the replication study I was able to ascertain whether the authors of the replication deemed their study a successful replication of the original. I also extracted from the original and replication, where possible, the statistical information (e.g., *t*-value and sample size) required to calculate an effect size (Cohen’s *d*). In cases where the original effect size differs from zero but the replicate does not, I used Simonsohn’s (2015) detectability approach to determine whether replication results are consistent with an effect size that was large enough to have been detectable in the original study. This approach rests on defining the effect size that would give the original study 33% power (*d*_33%_). A replication having an effect size significantly smaller than *d*_33%_ is inconsistent with the studied effect being big enough to have been detectable with the original sample size, which then casts doubt on the validity of the original finding (Simonsohn, 2015).

## Results and Discussion

### The number of replications in the literature

I found *n* = 11 papers that claimed to have replicated a previously published study (Table 1); however, one of these papers (Amos, 2009) re-analyzed the data of a previously published study (Møller & Cuervo, 2003) and another (Cath et al., 2008) did not replicate a specific study. Therefore, I found that only 0.023% (9/38730) of papers in *Ecology, Evolution, Behavior, and Systematics* journals claimed to truly replicate a previously published study.

**Table 1:** Replication studies found in full-text searches of (A) *Ecology, Evolution, Behavior, and Systematic* journals, and (B) *PeerJ*. The statistical significance of the studied effect (as judged by the author(s)) in both the original and replication are noted.

**Original study**	**Replication study**	**Significant effect in original study?**	**Claimed to have replicated original study?**	**Note**
**(a) Ecology, Evolution, Behaviour, and Systematics**				
Møller & Cuervo (2003)	Amos (2009)	Yes	No	Data re-analysis
No specific study	Cath et al. (2008)	Various	No claim made	
Cresswell et al. (2003)	Bulla et al. (2014)	Yes	No	Dependent (paired) data; could not calculate effect size
Keil & Ihssen (2004)	Keil, Ihssen & Heim (2006)	Yes	Yes	Insufficient data provided by Keil, Ihssen & Heim (2006) to calculate effect size
Range, Hentrup & Virányi (2011)	Müller et al. (2014)	Yes	No	Insufficient data provided by Range, Hentrup & Virányi (2011) to calculate effect size
Bentosela et al. (2009)	Riemer et al. (2016)	Yes	No	Dependent (paired) data; could not calculate effect size
Ringler et al. (2013)	Pasukonis et al. (2016)	Yes	Yes	Data available to calculate effect size for both studies
Jennings, Snook & Hoikkala (2014)	Ala-Honkola, Ritchie & Veltsos (2016)	No and yes	Yes and no	Data available to calculate effect size for both studies
Semm & Demaine (1986)	Ramírez et al. (2014)	Yes	No	Insufficient data to calculate effect sizes
Medina, Ávila & Morando (2013)	Troncoso-Palacios et al. (2015)	Yes	No	Insufficient data in Troncoso-Palacios et al. (2015) to calculate effect sizes
Steiner et al. (2010)	Burks, Mikó & Deans (2016)	Yes	No	Qualitative replication; no data to calculate effect size
**(b) PeerJ**				
Van Oystaeyen et al. (2014)	Holman (2014)	Yes	Yes	Insufficient data provided by Van Oystaeyen et al. (2014) to calculate effect size

Examination of *PeerJ* revealed one replication (Holman, 2014) out of 3,343 papers, giving a replication rate of 0.03%, a value that is nearly identical (*χ*^2^ = 6.7*e* − 29, *p* = 1, *df* = 1) to that observed in *Ecology, Evolution, Behavior, and Systematics* journals.

My analysis of the *Ecology, Evolution, Behavior, and Systematics* literature suggests that approximately 0.023% of studies in ecology and evolutionary biology truly replicated (or at least claimed to have) a previosuly published study. Although this rate is on par with other disciplines in the natural and social sciences (Makel, Plucker & Hegarty, 2012; Mueller-Langer et al., 2019; Evanschitzky et al., 2007; Reid, Soley & Winner, 1981) it is considerably lower than that reported by Kelly (2006) for the behavioural ecology literature. Kelly (2006) found that 25–34% of studies in behaviuoural ecology are partially/conceptually replicated while no studies were exactly replicated. I did not subdivide true replications in the current study and so direct comparisons with Kelly (2006) are not possible. However, the low replication rate observed in the current study might be due to an underestimation of conceptual replications if authors of this type of replication are less inclined to use the word “replication” in their paper (i.e., those studies would not have been highlighted here by text-mining). In other words, perhaps the bulk of the papers in Kelly (2006) that were categorized as partial/conceptual replications were conceptual and those types of studies were not identified here by my text-mining protocol.

I do not know of any study examining whether exact replications are more likely to be called a replication within the paper than a conceptual replication or the likelihhod that an author conducting a true replication will even label their study as such. It seems unlikely that an author who is explicitly conducting a replication, particualrly an exact replication, would not use the word “replication” somewhere in their article. However, perhaps authors are reticent to claim their study as a replication because the stigma of study replication persists and thus reduces publication success. Alternatively, perhaps the low rate observed here is due to a higher likelihood of replications being published in multidisciplinary biology journals rather than more targeted sources such as those in *Ecology, Evolution, Behavior, and Systematics*. Contrary to this prediction, I found no difference between the rate of publication in *PeerJ* (a multidisciplinary bioscience journal) and that in *Ecology, Evolution, Behavior, and Systematics* journals. Finally, perhaps the rate of replication observed here is not representative of the field as a whole because publication rates of replications might be higher in non-open access *Ecology, Evolution, Behavior, and Systematics* journals. This seems counter-intuitive, however, as anecdotal evidence suggests that open access journals are expected to have the highest likelihood of publishing a replication. Given these caveats it is important to emphasize that 0.023% likely underestimates the actual rate of study replication in ecology and evolution.

### The success of replications

The *n* = 10 replication studies (including Holman (2014) but not including Amos (2009); Cath et al., (2008)) yielded *n* = 11 effects (*n* = 2 effects in the Jennings, Snook & Hoikkala (2014)/Ala-Honkola, Ritchie & Veltsos (2016) replicate pair). Of these 11 effects, the replication authors concluded that their replication was successful in 36% of cases. I was able to calculate an effect size for both the original and replicate in only three cases; *n* = 1 for the Pasukonis et al. (2016)/Ringler et al. (2013) pair and *n* = 2 for the Jennings, Snook & Hoikkala (2014)/Ala-Honkola, Ritchie & Veltsos (2016) pair. In the other eight cases, both the replication and original were experiments with qualitative outcomes and did not record quantitative data, or either the original study or replication did not provide the data required to calculate an effect size (Table 1).

**Figure 1:**
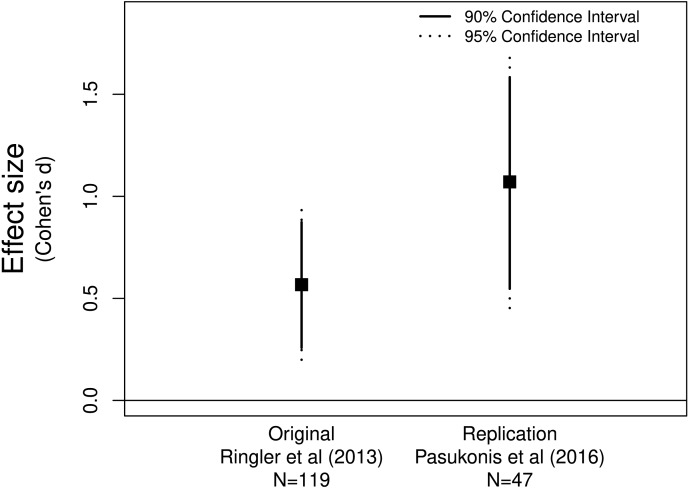
*Allobates femoralis* frog fathers anticipate distance to deposition site. The original (Ringler et al., 2013) and replicate (Pasukonis et al., 2016) studies both provide evidence for a positive effect greater than zero.

Pasukonis et al. (2016) replicated Ringler et al.’s (2013) study examining whether male *Allobates femoralis* frogs anticipate the distance to tadpole deposition sites. Pasukonis et al. (2016) declared that their replication study was successful since they found a significant positive correlation between distance traveled and tadpole number, the same finding as Ringler et al. (2013). Assessing the effect sizes and confidence intervals supports their conclusion as both effect sizes were positive and neither of the confidence intervals included zero (Fig. 1). These data suggest that father frogs can indeed anticipate the distance they need to travel to deposit their offspring.

**Figure 2:**
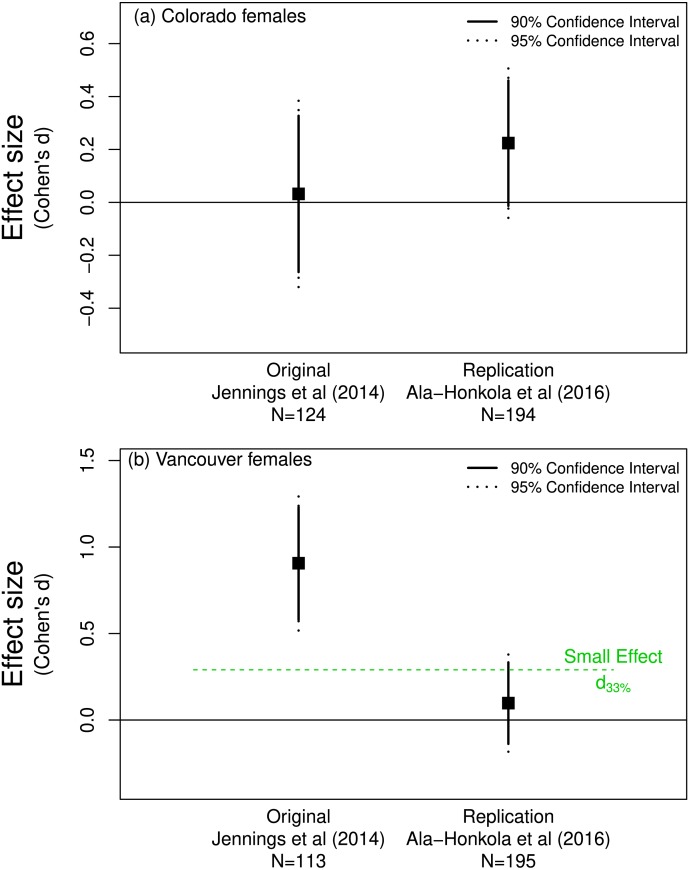
Mate preference of female *Drosophila montana* from (A) Colorado for males from Colorado or Vancouver, and from (B) Vancouver for males from Colorado or Vancouver. (A) shows that both the original (Jennings, Snook & Hoikkala, 2014) and replicate (Ala-Honkola, Ritchie & Veltsos, 2016) studies support a lack of preference by Colorado females since both effects overlap zero. The effect of the replicate in (B) does not differ from *d*_33%_ and so does not refute the claim in the original paper that Vancouver females show a mate preference.

Jennings, Snook & Hoikkala (2014) found little evidence of pre-mating isolation in populations of *Drosophila montana*. They found that females from Colorado accepted as mates males from Colorado and Vancouver with equal probability. Ala-Honkola, Ritchie & Veltsos (2016) found the same result (based on *p*-values) in their replication study. Figure 2A supports this conclusion as the effect sizes for both the original and replication overlap zero.

In contrast, Jennings, Snook & Hoikkala (2014) found that Vancouver females were more discriminating: they preferred to mate with males from Vancouver rather than those from Colorado. Ala-Honkola, Ritchie & Veltsos (2016) concluded that they did not replicate this finding, instead concluding that Vancouver females showed no significant mate preference one way or the other (Fig. 2B). Although the confidence interval for the replicated effect overlapped zero, a one-sided test for the effect being equal to *d*_33%_ = 0.29 (*n* = 113 thus *n* = 56.5 per group) was not rejected (*p* = 0.09). This is consistent with the notion that the effect found by Jennings, Snook & Hoikkala (2014) in the original study was indeed detectable given their sample size or in Simonsohn’s parlance their “telescope” was sufficiently large. Importantly, this suggests that the effect discovered by Jennings, Snook & Hoikkala (2014) might be biologically important and worthy of further investgation. Simonsohn’s (2015) detectability approach suggests that Ala-Honkola, Ritchie & Veltsos’s (2016) replication is in line with Jennings, Snook & Hoikkala’s (2014) original finding and was not a replication failure as Ala-Honkola, Ritchie & Veltsos (2016) reported in their paper.

## Conclusion

This formal analysis of three replicated effect sizes shows that the replicate study supported the original conclusion in two-thirds of cases. This sample size is too small to support sweeping generalizations of the efficacy of replications in *Ecology, Evolution, Behavior, and Systematics*; however, that authors provided the relevant information to calculate an effect size in only three of 11 cases is cause for concern. I recommend that future authors either include a detectability analysis (Simonsohn, 2015) as part of their replication study or at least provide the information required for its calculation.

##  Supplemental Information

10.7717/peerj.7654/supp-1 Supplemental Information 1 Rate and success of study replication in ecology and evolution

## Additional Information and Declarations

### Data Availability

The following information was supplied regarding data availability: Data, Python code, and R code are available at the Open Science Framework (OSF): Kelly, Clint D. 2019. “Replication in Ecology and Evolution.” OSF. July 20. 10.17605/OSF.IO/WR286.

## References

[ref-1] Ala-Honkola O, Ritchie MG, Veltsos P (2016). Postmating–prezygotic isolation between two allopatric populations of *Drosophila montana*: fertilisation success differs under sperm competition. Ecology and Evolution.

[ref-2] Amos W (2009). Sexual selection does not influence minisatellite mutation rate. BMC Evolutionary Biology.

[ref-3] Begley CG, Ellis LM (2012). Raise standards for preclinical cancer research. Nature.

[ref-4] Bentosela M, Jakovcevic A, Elgier AM, Mustaca AE, Papini MR (2009). Incentive contrast in domestic dogs (*Canis familiaris*). Journal of Comparative Psychology.

[ref-5] Brandt MJ, IJzerman H, Dijksterhuis A, Farach FJ, Geller J, Giner-Sorolla R, Grange JA, Perugini M, Spies JR, Van ‘t Veer A (2014). The replication recipe: what makes for a convincing replication?. Journal of Experimental Social Psychology.

[ref-6] Bulla M, Cresswell W, Rutten AL, Valcu M, Kempenaers B (2014). Biparental incubation-scheduling: no experimental evidence for major energetic constraints. Behavioral Ecology.

[ref-7] Burks KN, Mikó I, Deans AR (2016). Dendrocerus mexicali (Hymenoptera, Ceraphronoidea, Megaspilidae): novel antennal morphology, first description of female, and expansion of known range into the US. Zookeys.

[ref-8] Camerer C, Dreber A, Forsell E, Ho T, Huber J, Johannesson M, Kirchler M, Almenberg J, Altmejd A, Chan T, Heikensten E, Holzmeister F, Imai T, Isaksson S, Nave G, Pfeiffer T, Razen M, Wu H (2016). Evaluating replicability of laboratory experiments in economics. Science.

[ref-9] Cath D, Van Grootheest D, Willemsen G, Van Oppen P, Boomsma D (2008). Environmental factors in obsessive-compulsive behavior: evidence from discordant and concordant monozygotic twins. Behavior Genetics.

[ref-10] Cresswell W, Holt S, Reid JM, Whitfield DP, Mellanby RJ (2003). Do energetic demands constrain incubation scheduling in a biparental species. Behavioral Ecology.

[ref-11] Cumming G (2008). Replication and p intervals: p values predict the future only vaguely, but confidence intervals do much better. Perspectives on Psychological Science.

[ref-12] Evanschitzky H, Armstrong JS (2010). Replications of forecasting research. International Journal of Forecasting.

[ref-13] Evanschitzky H, Baumgarth C, Hubbard R, Armstrong JS (2007). Replication research’s disturbing trend. Journal of Business Research.

[ref-14] Fidler F, Chee YE, Wintle BC, Burgman MA, McCarthy MA, Gordon A (2017). Metaresearch for evaluating reproducibility in ecology and evolution. BioScience.

[ref-15] Fraser H, Parker T, Nakagawa S, Barnett A, Fidler F (2018). Questionable research practices in ecology and evolution. PLOS ONE.

[ref-16] Head M, Holman L, Lanfear R, Kahn A, Jennions M (2015). The extent and consequences of p-hacking in science. PLOS Biology.

[ref-17] Holman L (2014). Bumblebee size polymorphism and worker response to queen pheromone. PeerJ.

[ref-18] Hubbard R, Armstrong JS (1994). Replications and extensions in marketing: rarely published but quite contrary. International Journal of Research in Marketing.

[ref-19] Hubbard R, Vetter DE (1991). Replications in the finance literature: an empirical study. Quarterly Journal of Business and Economics.

[ref-20] Hubbard R, Vetter DE (1996). An empirical comparison of published replication research in accounting, economics, finance, management, and marketing. Journal of Business Research.

[ref-21] Ioannidis JPA (2005). Contradicted and initially stronger effects in highly cited clinical research. Journal of the American Medical Association.

[ref-22] Iorns E (2013). Reproducibility initiative receives $1.3 M grant to validate 50 landmark cancer studies. Center for open science.

[ref-23] Jennings JH, Snook RR, Hoikkala A (2014). Reproductive isolation among allopatric Drosophila montana populations. Evolution.

[ref-24] Keil A, Ihssen N (2004). Identification facilitation for emotionally arousing verbs during the attentional blink. Emotion.

[ref-25] Keil A, Ihssen N, Heim S (2006). Early cortical facilitation for emotionally arousing targets during the attentional blink. BMC Biology.

[ref-26] Kelly CD (2006). Replicating empirical research in behavioral ecology: how and why it should be done but rarely ever is. The Quarterly Review of Biology.

[ref-27] Klein R, Vianello M, Hasselman F, Adams BG, Adams Jr RB, Alper S, Vega D, Aveyard M, Axt J, Babaloia M, Bahnk Š, Berkics M, Bernstein MJ, Berry DR, Bialobrzeska O, Bocian K, Brandt M, Busching R, Cai H, Cambier F, Cantarero K, Carmichael CL, Cemalcilar Z, Chandler JJ, Chang J-H, Chatard A, Chen E, Cheong W, Cicero DC, Coen S, Coleman JA, Collisson B, Conway M, Corker KS, Curran PG, Cushman F, Dalgar I, Davis WE, De Bruijn M, De Vries M, Devos T, Doǧulu C, Dozo N, Dukes K, Dunham Y, Durrheim K, Easterbrook M, Ebersole CR, Edlund J, English AS, Eller A, Finck C, Freyre MÁ, Frankowska N, Friedman M, Galliani EM, Ghoshal T, Giessner SR, Gill T, Gnambs T, Gonzalez R, Graham J, Grahe J, Grahek I, Green E, Hai K, Haigh M, Haines EL, Hall MP, Heffernan ME, Hicks JA, Houdek P, Van der Hulst M, Huntsinger JR, Huynh HP, IJzerman H, Inbar Y, Innes-Ker A, Gomez A, John M-S, Jimenez-Leal W, Joy-Gaba J, Kamiloglu R, Kappes A, Kappes H, Karabati S, Karick H, Keller VN, Kende A, Kervyn N, Knezevic G, Kovacs C, Krueger LE, Kurapov G, Kurtz J, Lakens D, Lazarevic L, Levitan C, Lewis Jr N, Lins S, Maassen E, Maitner A, Malingumu W, Mallett R, Marotta S, McIntyre J, Medjedovic J, Milfont TL, Morris W, Myachykov A, Murphy S, Neijenhuijs KI, Nelson AJ, Neto F, Nichols AL, O’Donnell SL, Oikawa M, Orosz G, Osowiecka M, Packard G, Pérez R, Petrovic B, Pilati R, Pinter B, Podesta L, Pollmann M, Rosa AD, Rutchick AM, Patricio Saavedra M, Sacco A, Saeri AK, Salomon E, Schmidt K, Schönbrodt F, Sekerdej M, Sirlopu DR, Skorinko J, Smith MA, Smith-Castro V, Sobkow A, Sowden WJ, Spachtholz P, Steiner TG, Stouten J, Street CN, Sundfelt O, Szumowska E, Tang A, Tanzer NK, Tear M, Theriault J, Thomae M, Torres D, Traczyk J, Tybur JM, Ujhelyi A, Van Assen MALM, Van ’t Veer A, Echeverría AV, Vaughn LA, Vźquez A, Verniers C, Verschoor M, Voermans I, Vranka M, Welch C, Wichman A, Williams LA, Woodzicka JA, Wronska MK, Young L, Zelenski JM, Nosek BA (2018). Many Labs 2: Investigating variation in replicability across samples and settings. Advances in Methods and Practices in Psychological Science.

[ref-28] Klein RA, Ratliff KA, Vianello M, Adams Jr RB, Bahník S, Bernstein MJ, Bocian K, Brandt MJ, Brooks B, Brumbaugh CC (2014). Investigating variation in replicability. Social Psychology.

[ref-29] Lykken DT (1968). Statistical significance in psychological research. Psychological Bulletin.

[ref-30] Makel M, Plucker J, Hegarty B (2012). Replications in psychology research: how often do they really occur?. Perspectives on Psychological Science.

[ref-31] Makel MC, Plucker JA (2014). Facts are more important than novelty: replication in the education sciences. Educational Researcher.

[ref-32] Medina CD, Ávila LJ, Morando M (2013). Hacia una Taxonomía Integral: poniendo a prueba especies candidatas relacionadas a Liolaemus buergeri Werner 1907 (Iguania: Liolaemini) mediante análisis morfológicos. Cuadernos de herpetología.

[ref-33] Møller A, Cuervo J (2003). Sexual selection, germline mutation rate and sperm competition. BMC Evolutionary Biology.

[ref-34] Müller CA, Riemer S, Virányi Z, Huber L, Range F (2014). Dogs learn to solve the support problem based on perceptual cues. Animal Cognition.

[ref-35] Mueller-Langer F, Fecher B, Harhoff D, Wagner GG (2019). Replication studies in economics—how many and which papers are chosen for replication, and why. Research Policy.

[ref-36] Nakagawa S, Parker TH (2015). Replicating research in ecology and evolution: feasibility, incentives, and the cost-benefit conundrum. BMC Biology.

[ref-37] Open Science Collaboration (2015). Estimating the reproducibility of psychological science. Science.

[ref-38] Palmer AR (2000). Quasi-replication and the contract of error: lessons from sex ratios, heritabilities and fluctuating asymmetry. Annual Review of Ecology and Systematics.

[ref-39] Parker T, Forstmeier W, Koricheva J, Fidler F, Hadfield J, Chee Y, Kelly C, Gurevitch J, Nakagawa S (2016). Transparency in ecology and evolution: real problems, real solutions. Trends in Ecology & Evolution.

[ref-40] Pasukonis A, Trenkwalder K, Ringler M, Ringler E, Mangione R, Steininger J, Warrington I, Hödl W (2016). The significance of spatial memory for water finding in a tadpole-transporting frog. Animal Behaviour.

[ref-41] Prinz F, Schlange T, Asadullah K (2011). Believe it or not: how much can we rely on published data on potential drug targets. Nature Reviews Drug Discovery.

[ref-42] Ramírez E, Marín G, Mpodozis J, Letelier JC (2014). Extracellular recordings reveal absence of magneto sensitive units in the avian optic tectum. Journal of Comparative Physiology A: Neuroethology, Sensory, Neural, and Behavioral Physiology.

[ref-43] Range F, Hentrup M, Virányi Z (2011). Dogs are able to solve a means-end task. Animal Cognition.

[ref-44] Reid LN, Soley LC, Winner RD (1981). Replication in advertising research: 1977, 1978, 1979. Journal of Advertising.

[ref-45] Riemer S, Ellis SL, Ryan S, Thompson H, Burman OH (2016). A reappraisal of successive negative contrast in two populations of domestic dogs. Animal Cognition.

[ref-46] Ringler E, Pasukonis A, Hödl W, Ringler M (2013). Tadpole transport logistics in a Neotropical poison frog: indications for strategic planning and adaptive plasticity in anuran parental care. Frontiers in Zoology.

[ref-47] Schmidt S (2009). Shall we really do it again? The powerful concept of replication is neglected in the social sciences. Review of General Psychology.

[ref-48] Semm P, Demaine C (1986). Neurophysiological properties of magnetic cells in the pigeon’s visual system. Journal of Comparative Physiology A.

[ref-49] Simons DJ (2014). The value of direct replication. Perspectives on Psychological Science.

[ref-50] Simonsohn U (2015). Small telescopes: detectability and the evaluation of replication results. Psychological Science.

[ref-51] Steiner SM, Kropf C, Graber W, Nentwig W, Klopfstein S (2010). Antennal courtship and functional morphology of tyloids in the parasitoid wasp Syrphoctonus tarsatorius (Hymenoptera: Ichneumonidae: Diplazontinae). Arthropod Structure & Development.

[ref-52] Troncoso-Palacios J, Díaz HA, Esquerré D, Urra FA (2015). Two new species of the Liolaemuselongatus-kriegi complex (Iguania, Liolaemidae) from Andean highlands of southern Chile. Zookeys.

[ref-53] Van Oystaeyen A, Oliveira RC, Holman L, Van Zweden JS, Romero C, Oi CA, d’Ettorre P, Khalesi M, Billen J, Wäckers F (2014). Conserved class of queen pheromones stops social insect workers from reproducing. Science.

[ref-54] Zwaan R, Etz A, Lucas R, Donnellan M (2017). Making replication mainstream. Behavioral and Brain Sciences.

